# Evolution of risk assessment strategies for food and feed uses of stacked GM events

**DOI:** 10.1111/pbi.12551

**Published:** 2016-03-28

**Authors:** Catherine Kramer, Phil Brune, Justin McDonald, Monique Nesbitt, Alaina Sauve, Sabine Storck‐Weyhermueller

**Affiliations:** ^1^ Syngenta Crop Protection LLC. Research Triangle Park NC USA; ^2^ Syngenta Crop Protection AG Basel Switzerland

**Keywords:** genetically modified, stacks, biotechnology, risk assessment, problem formulation

## Abstract

Data requirements are not harmonized globally for the regulation of food and feed derived from stacked genetically modified (GM) events, produced by combining individual GM events through conventional breeding. The data required by some regulatory agencies have increased despite the absence of substantiated adverse effects to animals or humans from the consumption of GM crops. Data from studies conducted over a 15‐year period for several stacked GM event maize (*Zea mays* L.) products (Bt11 ×  GA21, Bt11 ×  MIR604, MIR604 ×  GA21, Bt11 ×  MIR604 ×  GA21, Bt11 ×  MIR162 ×  GA21 and Bt11 ×  MIR604 ×  MIR162 ×  GA21), together with their component single events, are presented. These data provide evidence that no substantial changes in composition, protein expression or insert stability have occurred after combining the single events through conventional breeding. An alternative food and feed risk assessment strategy for stacked GM events is suggested based on a problem formulation approach that utilizes (i) the outcome of the single event risk assessments, and (ii) the potential for interactions in the stack, based on an understanding of the mode of action of the transgenes and their products.

## Introduction

Genetically modified (GM) crops have been widely adopted since commercial release more than 20 years ago, with a steady growth each year in biotech hectarage planted globally (James, 2014). The benefits of GM crops were recently documented by Klumper and Qaim (2014) and include an overall increase in yield of almost 22%, a 36.9% reduction in pesticide use and an increase in farmer profit slightly over 68%. GM crops are increasingly being exploited globally, benefiting 18 million farmers in 28 countries in 2014 (James, 2014).

### What is a GM stack?

Crop productivity has undergone continuous improvement by conventional plant breeding since crop plants were originally domesticated for agricultural purposes. For example, over 100 genes conferring beneficial traits from wild relatives have been incorporated into the genomes of 19 of the world's major crops by conventional breeding, creating new varieties with disease resistance, abiotic stress resistance and improved quality (Hajjar and Hodgkin, 2007).

Conventional breeding techniques can also be used to combine traits from two or more GM events. A GM event is defined as the insertion of DNA into the plant genome as a result of a single transformation process (Pilacinski *et al*., 2011). Individual events may contain one or more transgenes, and these events are sometimes referred to as molecular stacks (Que *et al*., 2010). Alternatively, using conventional breeding to combine GM events does not involve insertion of new recombinant DNA sequence into the genome and does not modify the existing genomic DNA, so stacked GM events are, by definition, not a new event (Pilacinski *et al*., 2011). The data presented in this study include analysis of stacked GM events created by conventional breeding of single events developed by Syngenta and will be referred to as stacked GM events or stacks.

### Why are stacked events important?

The ability to offer multiple benefits in a single product have resulted in many GM events being combined by conventional breeding techniques, offering combinations of insect resistance and herbicide tolerance genes. These types of stacks are also referred to as ‘pyramids’, characterized by multiple traits that confer resistance to a specific pest or pest complex. For example, Syngenta's Agrisure Viptera^®^ 3111 stack provides control of 14 insect pests and tolerance to both glyphosate and glufosinate herbicides.^1^ GM events combining different modes of action for protection against specific insect(s) pests are a valuable strategy for management of insect resistance.

### How are single GM events assessed for risk?

The principles underpinning risk assessments of GM events have been well described by the Food and Agricultural Organization/World Health Organization (FAO/WHO 1996, 2000), the Organization for Economic Cooperation and Development (OECD 2010), the Codex Alimentarius (2003, 2009) and by national regulatory agencies (EC 2013, EFSA 2011a, EPA 2014, FDA 1992, Health Canada 2006). Most commercial GM crops express transgenes, which encode proteins that confer the desired traits, for example pest control or herbicide tolerance. Many of these transgenes are derived from other plants or microorganisms ubiquitous and/or abundant in the environment and food and feed chain. Regardless of the nature and origin of the transgenes, newly developed GM events undergo comprehensive safety assessments prior to commercial release. The history of safe use of the conventional crop, which has been consumed for decades, can be used as a baseline for the safety assessment of new GM plant varieties (Kok and Kuiper, 2003).

Data generated for a risk assessment of a new GM single event intended for food and feed typically includes (i) molecular characterization of the genetic insertion, (ii) bioinformatic assessment of the inserted sequences for similarity to known allergens or toxins, (iii) protein toxicity studies, (iv) analysis of the physicochemical properties of the introduced proteins as they pertain to stability and activity, (v) quantitation of the proteins expressed by the transgenes *in planta*, (vi) nutritional equivalence assessed by compositional analysis and animal feeding studies, and (vii) an estimation of human dietary exposure. Although individual regulatory agencies may have different specific requirements, these data meet the requirements of most global regulatory agencies in regions that import food and feed commodities.

The molecular characterization of a GM event includes verifying the sequence of the inserted DNA and the host genome flanking sequences and confirmation of the copy number of the inserted sequences. Correct position of the promoter and terminator sequences with respect to the coding sequence(s) confirms that only the intended transcripts(s) and protein(s) are encoded by the transgene(s). The flanking sequence is used to assess the possible disruption of endogenous genes in the host plant and the potential generation of new open reading frames.

Proteins are an essential component of both human and animal diets, and most are safe to consume. But all known food allergens are proteins and there are also rare examples of proteins that are toxic to animals, including humans. The potential for proteins expressed by the transgenes to be allergenic or toxic is assessed using comparative bioinformatic data to identify similarities in sequence to known allergens or toxins and by characterization of physicochemical properties consistent with proteinaceous allergens and toxins.

Protein expression studies are conducted to generate a profile of transgenic protein expression across the life cycle of the crop produced under field conditions. These data can be used to estimate potential human and livestock exposure to the transgenic protein present in food and feed products and for determining appropriate dose levels for protein toxicity studies. Protein toxicity studies often use rats or mice to evaluate the effects of direct exposure to transgenic proteins administered via gavage, based on the recommended limit dose (OECD 2001) or the theoretical exposure level (OECD 2008).

The major objective of compositional equivalence evaluations of new GM crop varieties is to assess whether the new GM event is comparable in nutrient and antinutrient content to the conventional crop that already has an established history of safe consumption.

In addition to compositional equivalence studies, some regulatory agencies require animal feeding studies with diets prepared from the raw agricultural commodity originating from the GM crop to further assess nutritional value and wholesomeness.

### How are stacked events assessed for safety?

When a GM stack is created by conventional breeding, the inserted DNA from each individual event is transferred from parent to progeny similarly to that for endogenous genes. There is no additional recombinant DNA sequences introduced when stacking GM events by conventional breeding. For the purposes of this discussion on food and feed risk assessment of GM stacks, it is assumed that the single events that comprise the stack have been individually and comprehensively evaluated for food and feed safety, as described above, and that the novel proteins expressed in the GM stack have been shown to pose negligible risk to human and animal health.

Currently, there is no global consensus for the regulation of previously approved GM events combined by conventional breeding (Pilacinski *et al*., 2011). Consequently, some regulatory agencies require no additional safety data, while others require a repeat of studies already conducted for the safety assessments of the individual events. Still others have chosen to regulate only the GM stacks, which are inclusive of the greatest number of events in the stacks under consideration (‘higher‐order stacks’, Pilacinski *et al*., 2011; Ramjoue, 2008). Countries that have adopted policies whereby approval of a higher‐order stack may cover lower‐order stacks containing the same events include Argentina, Paraguay, Uruguay, Philippines, the European Union and Brazil (e.g. EC 2013, EFSA 2011a, Philippines 2013).

For countries that require additional data for food and feed approval of stacked GM events, these requirements may include confirmation of copy number and intactness of the inserted sequence contributed by each of the single events, verification that expression of the introduced genes is consistent with that of the single events, and nutritional equivalence studies. Recently, a new requirement to confirm transgenic insert stability by resequencing of the individual single events in stacked GM events has been implemented for import approval in EU member countries (EC 2013).

Compositional equivalence, protein expression and molecular characterization (insert stability and intactness) data generated for food and feed safety assessments of stacked GM events and the component single events are described for stacks containing two, three and four Syngenta events in six stacked event combinations (Bt11 × GA21, Bt11 × MIR604, MIR604 × GA21, Bt11 × MIR604 × GA21, Bt11 × MIR162 × GA21 and Bt11 × MIR604 × MIR162 × GA21). Data from individual studies were compiled to create a compendium representing the range of variation in composition and expression of single events and stacks across many years of agricultural production in different environments and in different germplasm. As expected, both environment and germplasm had an impact on composition and expression, but there were no indications that stacking the events had either an additive or synergestic effect. These data, together with molecular studies demonstrating insert stability, provide compelling evidence that when there is no likely interaction between the single event, extrapolation of single event risk assessment data to the stacked GM events is warranted.

An understanding of the modes of action of the individual transgenes, the products they produce and the transgenic protein expression profile can be used with a problem formulation approach to identify potential interactions between the transgenes and transgene products contained in the combined events, on a case‐by‐case basis. When there is a likelihood of interaction, additional studies can be designed to address anticipated interaction(s) and to either confirm or update the existing safety assessments.

## Results

### Compositional analysis

For each nutritional component, the mean levels for the single events and the stacks were very similar (Table 1). The compositional analysis data for Bt11 was limited, so no comparison with that single event was possible for many of the analytes measured in the stacks. The range of values for each of the six stacked event hybrids were compared to the compiled prediction intervals derived from data of each of the corresponding single events contained in each stack. For example, the range of protein levels in the Bt11 × MIR162 × GA21 stack (10.0–12.7% dry weight) was compared to the range of the prediction intervals for Bt11, MIR162 and GA21 (6.87%–13.5%). Most values of the stacks were within the collective prediction interval ranges for the corresponding singles. When a low or high value for a stack was not within the prediction intervals for the singles, it was often due to only one or a few of the stack values falling outside of the prediction intervals.

**Table 1:** Levels of nutritional and antinutritional components in grain from single event and stacked event maize

Event or stack	Bt11	GA21	MIR604	MIR162	Bt11 MIR162 MIR604 GA21	Bt11 MIR162 GA21	Bt11 MIR604 GA21	Bt11 MIR604	Bt11 GA21	MIR604 GA21
*N* =	21^a^	53^b^	52	34^c^	18	18	36^d^	18^e^	36	18^f^
Protein (%)										
Mean	8.59	10.8	10.8	9.85	10.7	10.9	10.3	9.88	10.5	11.1
SD	0.8055	1.336	1.111	0.8663	0.8052	0.7492	1.279	0.8326	1.446	0.9609
Min	7.30	6.7	7.96	7.50	9.40	10.0	7.39	7.50	7.78	9.3
Max	10.6	13.7	13.3	11.2	12.5	12.7	13.3	10.8	13.0	12.5
Prediction interval	6.87–10.3	8.06–13.5	8.50–13.0	8.06–11.6						
Fat (%)										
Mean	3.2	4.0	3.9	3.7	4.5	4.2	4.3	3.8	4.4	4.6
SD	0.433	0.540	0.420	0.340	0.304	0.423	0.436	0.335	0.538	0.422
Min	2.5	2.9	2.9	3.3	4.0	3.2	3.3	3.3	3.2	3.9
Max	4.1	4.9	4.7	4.6	**5.2**	5.0	5.1	4.3	**5.4**	**5.3**
Prediction interval	2.3–4.1	2.9–5.1	3.1–4.8	3.0–4.4						
Carbohydrates (%)										
Mean	–	83.8	83.8	84.9	83.3	83.4	83.9	84.9	83.5	82.6
SD	–	1.576	1.260	0.9564	0.9775	0.9192	1.477	0.9694	1.673	1.113
Min	–	81.0	80.5	83.2	81.2	80.7	80.7	83.7	80.9	81.0
Max		87.8	86.6	87.1	85.3	84.3	86.8	**87.0**	86.3	84.8
Prediction interval	–	80.6–87.0	81.2–86.4	83.0–86.9						
Acid detergent fibre (%)										
Mean	–	3.7	4.6	4.4	3.6	3.5	3.6	4.8	5.0	5.8
SD	–	0.577	1.16	1.02	0.493	0.631	0.372	0.910	1.44	0.756
Min	–	2.6	2.7	3.2	2.7	2.7	*2.4*	3.2	3.3	4.9
Max		5.4	7.7	7.0	4.5	5.5	4.4	6.1	**8.4**	7.5
Prediction interval	–	2.6–4.9	2.3–7.0	2.3–6.5						
Neutral detergent fibre (%)										
Mean	–	11.0	12.0	11.7	9.70	11.2	10.2	11.5	11.4	13.0
SD	–	1.761	1.861	1.148	0.9773	2.061	1.043	0.7710	0.9503	1.189
Min	–	7.50	9.38	10.0	7.70	8.80	*7.20*	9.90	9.62	10.9
Max		14.4	15.5	15.1	11.9	**18.1**	12.7	13.2	13.4	15.5
Prediction interval	–	7.47–14.6	8.19–15.7	9.34–14.1						
Starch (%)										
Mean	72.7	61.3	60.9	66.3	62.0	66.3	67.8	65.0	66.0	59.6
SD	0.9532	5.147	7.275	4.302	6.224	6.186	4.919	2.089	5.355	2.906
Min	70.8	46.8	51.4	54.8	48.0	58.5	53.8	61.5	55.3	55.1
Max	74.3	71.2	73.4	73.1	69.5	**78.4**	75.6	69.2	**74.9**	64.7
Prediction interval	70.6–74.7	50.9–71.8	46.1–75.6	57.4–75.2						
Calcium (ppm)										
Mean	–	51.1	47.9	46.4	47.7	44.4	50.1	39.7	54.0	45.2
SD	–	8.248	12.15	12.99	9.358	4.278	8.738	4.467	10.00	4.090
Min	–	37.3	30.5	29.4	34.4	38.7	38.7	31.7	39.2	37.5
Max		74.4	85.4	93.7	**76.2**	56.6	71.9	48.0	**85.2**	49.8
Prediction interval	–	34.4–67.9	23.2–72.5	19.5–73.2						
Copper (ppm)										
Mean	–	1.31	1.76	1.71	1.41	1.58	1.48	1.27	1.59	1.29
SD	–	0.1672	0.5628	0.4760	0.1813	0.1063	0.3616	0.3969	0.2436	0.09364
Min	–	0.980	1.00	0.960	1.01	1.40	1.05	0.950	1.21	1.17
Max		1.81	4.30	2.90	1.72	1.80	2.65	2.70	**2.20**	1.45
Prediction interval	–	0.971–1.65	0.623–2.90	0.732–2.70						
Iron (ppm)										
Mean	–	22.4	26.3	21.6	22.1	28.3	22.3	20.4	22.6	23.1
SD	–	4.149	4.758	2.926	1.708	1.739	3.568	1.971	3.904	2.440
Min	–	15.7	17.4	17.3	18.6	24.3	15.5	17.5	16.3	18.9
Max		42.3	44.5	33.4	25.5	**32.2**	**36.8**	24.0	**38.0**	27.2
Prediction interval	–	14.0–30.8	16.6–35.9	15.5–27.6						
Magnesium (ppm)										
Mean	–	1233	1220	1290	1283	1323	1232	1252	1351	1503
SD	–	117.97	125.00	102.62	94.980	96.881	126.60	117.39	195.75	85.392
Min	–	896	919	1090	1060	1150	*966*	1080	1030	1380
Max		1510	1480	1530	1420	**1540**	**1520**	**1490**	**1790**	**1660**
Prediction interval	–	994–1472	967–1473	1078–1502						
Manganese (ppm)										
Mean	–	6.68	5.63	6.38	6.30	6.61	5.64	6.20	6.55	7.40
SD	–	1.048	1.002	1.972	0.9441	0.8353	0.9827	1.093	1.607	1.413
Min	–	3.86	3.35	4.14	4.69	5.02	3.81	4.39	*4.48*	4.98
Max		8.87	7.93	16.2	8.22	8.13	7.57	**8.53**	**9.88**	**9.62**
Prediction interval	–	4.56–8.81	3.60–7.66	2.31–10.5						
Phosphorus (ppm)										
Mean	–	3295	3092	3034	3459	3543	3116	3127	3383	3993
SD	–	405.24	501.26	261.05	318.63	256.49	523.95	185.88	741.71	269.09
Min	–	2180	1660	2430	2610	2940	*1740*	2790	*1880*	3560
Max		4170	3710	3550	3850	4000	3980	3470	**4760**	**4400**
Prediction interval	–	2474–4115	2076–4108	2496–3573						
Potassium (ppm)										
Mean	–	3743	3879	3694	3988	3994	3967	3373	4182	4399
SD	–	380.36	360.00	474.25	215.39	236.47	305.74	162.62	340.87	149.96
Min	–	2920	3060	3160	3570	3570	3350	*3010*	3650	4120
Max		4720	4650	4930	4340	4400	**4670**	3610	**5020**	**4650**
Prediction interval	–	2973–4514	3150–4609	2715–4672						
Selenium (ppm)										
Mean	–	0.178	0.188	0.292	0.206	0.145	0.175	0.216	0.206	0.271
SD	–	0.09044	0.08153	0.1862	0.1248	0.1029	0.1157	0.1367	0.1509	0.1857
Min	–	<LOQ	<LOQ	<LOQ	0.0718	<LOQ	<LOQ	0.0569	<LOQ	<LOQ
Max		0.337	0.360	0.655	0.492	0.399	**0.501**	**0.444**	**0.625**	**0.607**
Predictioninterval	–	<LOQ–0.368	<LOQ–0.363	<LOQ–0.683						
Zinc (ppm)										
Mean	–	24.1	23.2	20.9	25.1	25.6	22.3	21.5	22.3	22.1
SD	–	3.163	3.702	2.354	3.084	3.598	3.868	1.899	3.150	1.708
Min	–	17.9	16.9	15.9	20.3	19.9	16.1	18.2	*17.2*	19.1
Max		31.0	31.2	24.4	**31.8**	**32.9**	29.7	25.4	28.5	25.4
Predictioninterval	–	17.7–30.5	15.7–30.7	16.0–25.8						
β–Carotene (mg/100 g)										
Mean	–	0.112	0.123	0.217	0.0950	0.113	0.0992	0.344	0.170	0.222
SD	–	0.04016	0.02362	0.07010	0.01792	0.01164	0.01882	0.02576	0.06540	0.01144
Min	–	0.0576	0.0841	0.120	0.0710	0.0912	0.0613	0.311	0.0692	0.201
Max		0.208	0.195	0.316	0.123	0.140	0.130	**0.409**	**0.257**	0.241
Prediction interval	–	0.0307–0.193	0.0723–0.362	0.0755–0.171						
Thiamine HCl (mg/100 g)										
Mean	–	0.38	0.45	0.42	0.43	0.46	0.43	0.41	0.42	0.42
SD	–	0.0643	0.0553	0.0388	0.0355	0.0354	0.0306	0.0255	0.0320	0.0321
Min	–	0.24	0.37	0.35	0.34	0.40	0.37	0.36	0.35	0.35
Max		0.48	0.59	0.49	0.49	0.53	0.49	0.47	0.48	0.48
Prediction interval	–	0.251–0.511	0.337–0.497	0.342–0.566						
Riboflavin (mg/100 g)										
Mean	–	0.193	0.157	0.183	0.225	0.203	0.229	0.164	0.215	0.223
SD	–	0.03331	0.03430	0.03571	0.03629	0.03784	0.03793	0.02942	0.04888	0.04291
Min	–	0.137	0.111	0.112	0.168	0.157	0.162	0.111	*0.107*	0.144
Max		0.339	0.245	0.257	**0.292**	**0.307**	**0.365**	0.228	**0.330**	**0.291**
Prediction interval	–	0.126–0.261	0.109–0.256	0.0875–0.227						
Niacin (mg/100 g)										
Mean	–	2.65	2.81	2.54	2.25	2.86	2.64	2.28	3.19	3.29
SD	–	0.7651	0.4087	0.2896	0.3358	0.3604	0.5290	0.1661	0.4157	0.6157
Min	–	1.55	2.18	2.11	1.45	2.13	1.58	2.02	2.23	2.53
Max		4.25	3.79	3.31	2.85	3.45	3.67	2.74	3.99	**4.54**
Prediction interval	–	1.10–4.20	1.94–3.14	1.98–3.64						
Pyridoxine HCl (mg/100 g)										
Mean	–	0.682	0.432	0.529	0.623	0.649	0.679	0.522	0.718	0.692
SD	–	0.09275	0.2557	0.07769	0.06852	0.08177	0.1039	0.05942	0.09809	0.05853
Min	–	0.508	0.218	0.385	0.480	0.554	0.503	0.436	0.518	0.586
Max		0.952	1.11	0.694	0.718	0.831	**0.927**	0.636	**0.902**	0.848
Prediction interval	–	0.494–0.870	0.369–0.690	<LOQ–0.950						
Folic acid (mg/100 g)										
Mean	–	0.0277	0.0484	0.0343	0.0288	0.0297	0.0328	0.0258	0.0400	0.0455
SD	–	0.004676	0.01549	0.01056	0.00715	0.009504	0.00872	0.006184	0.01548	0.01975
Min	–	0.0178	0.0232	0.0210	0.0209	0.0209	0.0206	0.0179	0.0217	0.0273
Max		0.0403	0.0813	0.0624	0.0437	0.0525	**0.0626**	0.0456	**0.0797**	**0.0821**
Prediction interval	–	0.0182–0.0372	0.0125–0.0561	0.0170–0.0798						
α–Tocopherol (mg/100 g)										
Mean	–	0.800	0.993	1.02	0.814	0.801	1.19	1.27	1.26	1.03
SD	–	0.1946	0.2536	0.2809	0.1471	0.1791	0.4741	0.1339	0.3360	0.1499
Min	–	0.436	<LOQ	0.619	<LOQ	0.504	<LOQ	1.06	0.850	0.838
Max		1.27	1.86	1.54	1.07	1.11	**2.18**	1.59	**2.06**	1.32
Prediction interval	–	0.406–1.19	<LOQ–1.60	0.472–1.51						
16:0 Palmitic (% of total)										
Mean	11.6	13.2	14.0	14.0	14.0	14.0	14.3	12.5	15.0	15.4
SD	0.9110	0.726	0.8947	1.403	0.4133	0.2578	0.6363	0.3123	0.5231	0.3634
Min	10.9	11.2	12.5	12.2	12.9	13.6	13.0	11.9	13.6	15.0
Max	13.5	14.5	15.9	16.6	14.5	14.7	15.5	12.9	**15.7**	16.1
Prediction interval	9.39–13.8	11.8–14.7	11.1–16.9	12.2–15.8						
18:0 Stearic (% of total)										
Mean	2.32	2.36	2.08	1.75	1.71	1.68	2.03	1.93	2.16	2.19
SD	0.2328	0.2248	0.2764	0.1507	0.1219	0.09912	0.4006	0.1671	0.2526	0.07253
Min	2.02	1.89	1.72	1.44	*1.47*	1.47	1.51	1.68	1.78	2.08
Max	2.70	2.90	2.77	1.99	1.88	1.82	**3.20**	2.26	2.79	2.30
Prediction interval	1.75–2.89	1.91–2.82	1.44–2.06	1.52–2.64						
Oleic (% of total)										
Mean	27.2	25.4	25.7	23.8	26.0	25.8	28.4	25.4	27.4	24.7
SD	1.277	2.595	2.502	2.151	1.972	2.108	2.487	0.9934	2.163	0.4615
Min	26.3	21.1	21.6	19.2	22.3	21.5	22.9	23.6	25.2	23.3
Max	30.3	29.1	30.6	26.6	28.9	28.8	**33.2**	26.5	**32.3**	25.3
Prediction interval	24.1–30.3	20.2–30.7	19.4–28.3	20.6–30.8						
18:2 Linoleic (% of total)										
Mean	57.7	56.4	56.3	57.6	55.8	55.9	52.6	57.3	52.7	55.0
SD	1.759	2.928	3.633	1.226	2.051	2.041	3.155	0.9154	2.422	0.5780
Min	53.2	52.1	48.8	55.9	52.5	52.6	*46.4*	56.3	*46.5*	54.1
Max	59.1	62.9	60.7	59.7	59.4	59.7	58.4	59.3	55.9	56.5
Prediction interval	53.4–62.0	50.4–62.3	55.1–60.1	48.9–63.6						
18:3 Linolenic (% of total)										
Mean	1.32	1.58	1.62	1.87	1.59	1.67	1.70	1.71	1.70	1.64
SD	0.2805	0.08545	0.1297	0.1323	0.1289	0.1273	0.1160	0.05098	0.08048	0.06526
Min	0.914	1.31	1.42	1.66	1.46	1.54	1.54	1.64	1.58	1.53
Max	1.69	1.79	1.87	2.26	1.95	*2.06*	2.06	1.82	1.85	1.76
Prediction interval	0.635–2.00	1.41–1.75	1.59–2.14	1.35–1.88						
Ferulic acid (ppm)										
Mean	–	1993	1937	2663	1846	1866	2005	2336	2177	2285
SD	–	242.38	252.44	157.72	160.78	169.91	259.01	155.95	229.02	148.53
Min	–	1310	1340	2380	1560	1560	1530	*2010*	1540	1990
Max		2440	2510	2990	2280	2120	2510	2590	**2620**	2520
Prediction interval	–	1502–2484	2337–2989	1425–2449						
p–Coumaric acid (ppm)										
Mean	–	189	170	224	136	123	201	124	239	214
SD	–	27.51	81.31	58.06	20.39	15.16	70.85	12.13	52.22	14.75
Min	–	140	63.3	148	87.3	*96.1*	82.7	104	178	193
Max		259	371	354	167	157	**338**	139	**363**	246
Prediction interval	–	133–244	5.05–335	104–344						
Inositol (ppm)										
Mean	–	2896	2728	2642	2742	2678	2664	2859	3024	3766
SD	–	651.93	490.63	479.75	420.43	441.2	432.94	402.21	751.4	382.32
Min	–	1940	1580	1600	2170	1660	1830	2190	1720	3320
Max		5540	4030	3530	3710	3240	3520	3400	**4900**	**4570**
Prediction interval	–	1576–4217	1652–3632	1733–3722						
Phytic acid (%)										
Mean	–	0.795	0.714	0.744	0.861	0.759	0.860	0.714	0.932	0.940
SD	–	0.1230	0.2597	0.08178	0.1089	0.1805	0.1753	0.0767	0.1562	0.1105
Min	–	0.491	<LOQ	0.556	0.631	0.208	0.512	0.546	0.600	0.750
Max		1.02	1.21	0.880	1.14	0.969	**1.15**	0.837	**1.19**	**1.20**
Prediction interval	–	0.546–1.04	<LOQ–0.913	0.187–1.24						
Trypsin inhibitor (TIU^g^/mg)										
Mean	–	2.80	2.81	3.46	1.96	1.79	2.59	2.90	3.10	3.11
SD	–	1.253	0.4411	0.9429	0.2796	0.3214	0.696	0.4931	0.297	0.2416
Min	–	<LOQ	2.06	2.27	1.50	1.34	1.42	2.18	2.51	2.62
Max		5.41	3.83	5.03	2.55	2.38	3.99	3.63	3.69	3.54
Prediction interval	–	0.259–5.34	1.51–5.41	1.91–3.70						

All values are on a dry weight basis. Each event and stack was grown in separate field trials. *N *= total number of observations, but the mean and standard deviation (SD) do not include observations below the level of quantitation (<LOQ). Bold values indicates one or more values above the combined prediction interval for all of the single events within the stack; Italic values indicates one or more values below the combined prediction interval.

a For starch, *N *=* *18 and for each fatty acid, *N *=* *9;

b For starch *N *=* *5;

c For iron *N *=* *33;

d For copper, *N *=* *35;

e For selenium, *N *=* *16;

f For selenium, *N *=* *12.

g TIU = trypsin inhibitor unit.

About half of the stack ranges with values outside of the prediction intervals occurred among the minerals, which are not metabolized by the plant but are instead absorbed from the surrounding environment and thus are heavily influenced by such environmental parameters as soil type, soil cation exchange capacity and fertilization regimes.

Across all nutritional components, the Bt11 × GA21 stack had the greatest number of values outside of the prediction intervals. The Bt11 and the GA21 events were both present in four other stacks, yet the occurrence of values outside of the prediction intervals was inconsistent compared to the Bt11 × GA21 stack (see highlighted range values, Table 3). If two events in a stack were interacting with one another to cause changes in composition, then that interaction should occur whenever those specific events were combined in a stack. However, examination of the means and ranges of other stacks containing these events showed no such pattern.

All values within the data set fell within the ranges of the International Life Sciences Crop Composition Database (ILSI 2014), which reports nutritional component levels for conventional crops.

The data for the single events and stacks were generated from plants grown in multiple environments (years, location and germplasm). The observed variability in grain composition can be attributed to these differences. It is well documented that both growing environment and germplasm have a strong influence on the composition of maize (Harrigan *et al*., 2007; Reynolds *et al*., 2005; Skogerson *et al*., 2010; Venkatesh *et al*., 2014). These results are consistent with the expectation that these events have little effect on composition, whether as single events or stacked with one another, and are a good representation of the variability in composition inherent in maize.

### Protein expression

**Figure 1:**
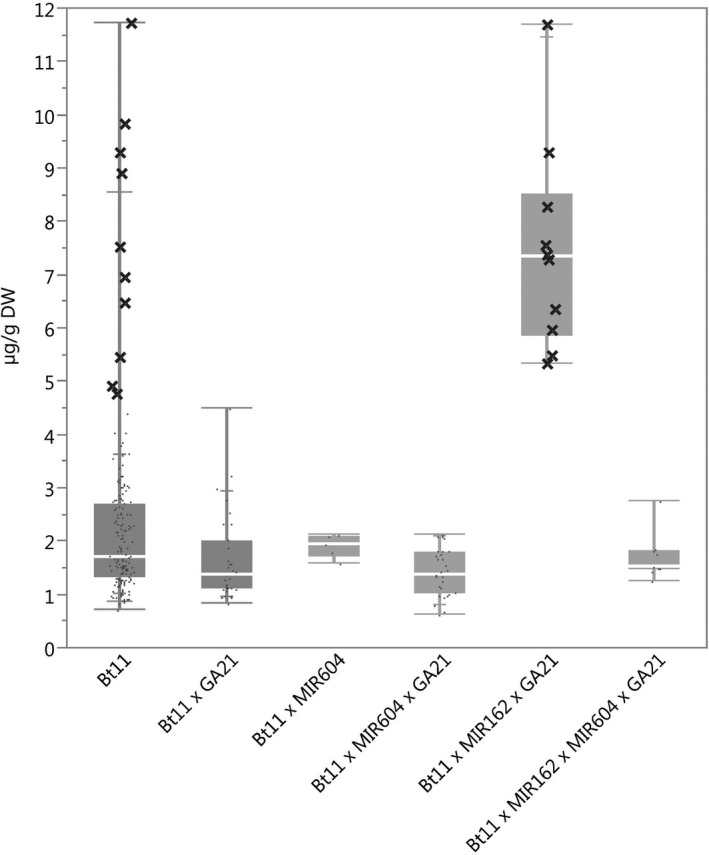
Cry1Ab concentrations in grain of event Bt11 (dark grey) and stacks containing event Bt11. *N *=* *170 (Bt11), 32 (Bt11 × GA21), 5 (Bt11 × MIR604), 29 (Bt11 × MIR604 × GA21), 10 (Bt11 × MIR162 × GA21) and 9 (Bt11 × MIR162 × MIR604 × GA21); Number of environments (combination of germplasm, year grown and location) = 11 (Bt11), 4 (Bt11 × GA21), 1 (Bt11 × MIR604), 3 (Bt11 × MIR604 × GA21), 1 (Bt11 × MIR162 × GA21), 1 (Bt11 × MIR162 × MIR604 × GA21); × = individual datapoints for Cry1Ab in event Bt11 and Bt11 × MIR162 × GA21 grown together in one field trial.

**Figure 2:**
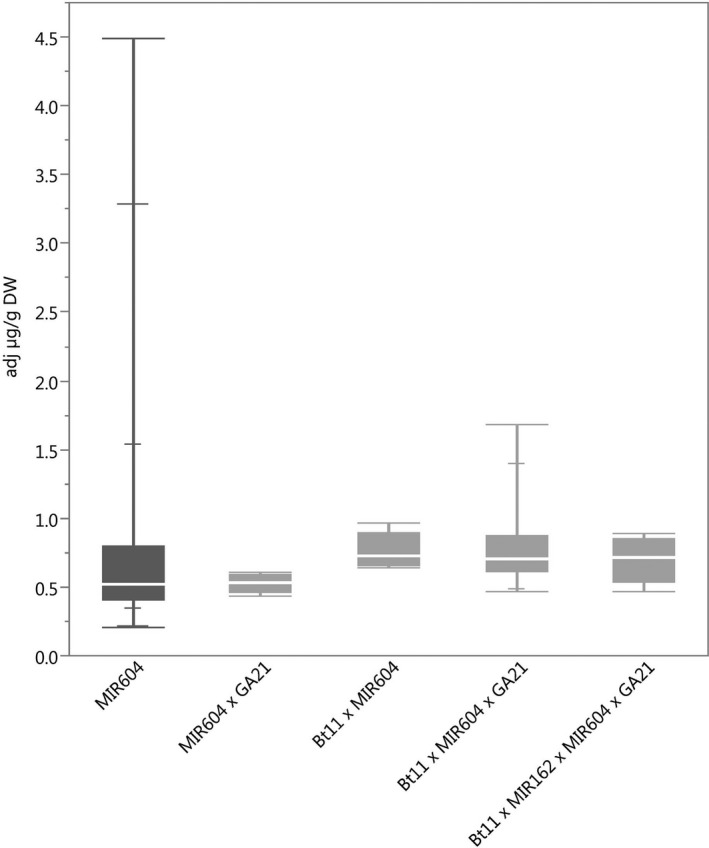
mCry3A concentrations in grain of event MIR604 (dark grey) and stacks containing event MIR604. *N *=* *101 (MIR604), 5 (MIR604 × GA21), 5 (Bt11 × MIR604), 19 (Bt11 × MIR604 × GA21) and 8 (Bt11 × MIR162 × MIR604 × GA21); Number of environments (combination of germplasm, year grown and location) = 7 (MIR604), 1 (MIR604 × GA21), 1 (Bt11 × MIR604), 3 (Bt11 × MIR604 × GA21) and 1 (Bt11 × MIR162 × MIR604 × GA21).

**Figure 3:**
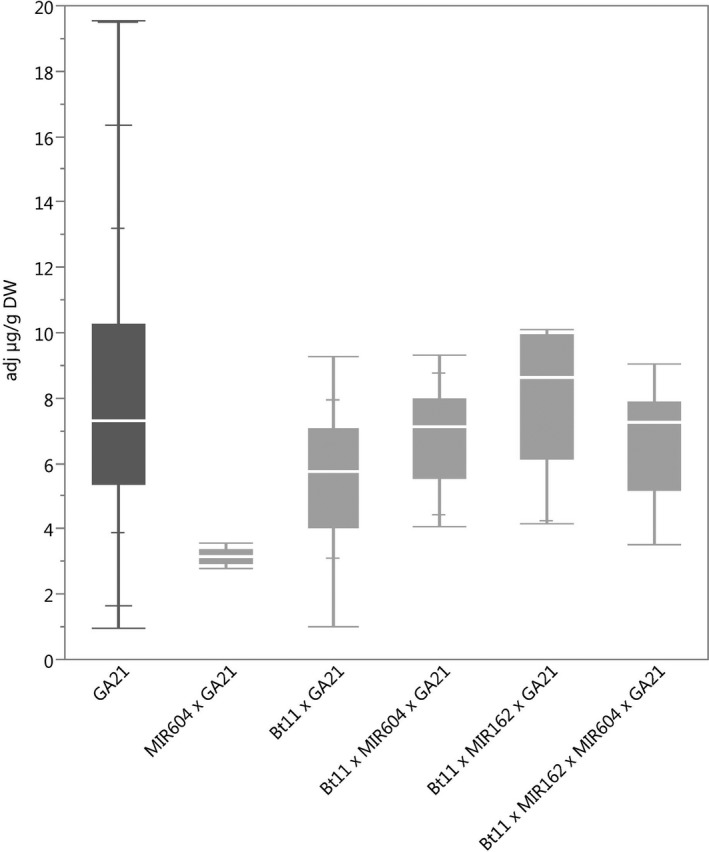
mEPSPS concentrations in grain of event GA21(dark grey) and stacks containing event GA21. *N *=* *208 (GA21), 5 (MIR604 × GA21), 30 (Bt11 × GA21), 29 (Bt11 × MIR604 × GA21), 10 (Bt11 × MIR162 × GA21) and 8 (Bt11 × MIR162 × MIR604 × GA21); Number of environments (combination of germplasm, year grown and location) = 12 (GA21), 1 (MIR604 × GA21), 4 (Bt11 × GA21), 3 (Bt11 × MIR604 × GA21), 1 (Bt11 × MIR162 × GA21) and 1 (Bt11 × MIR162 × MIR604 × GA21).

**Figure 4:**
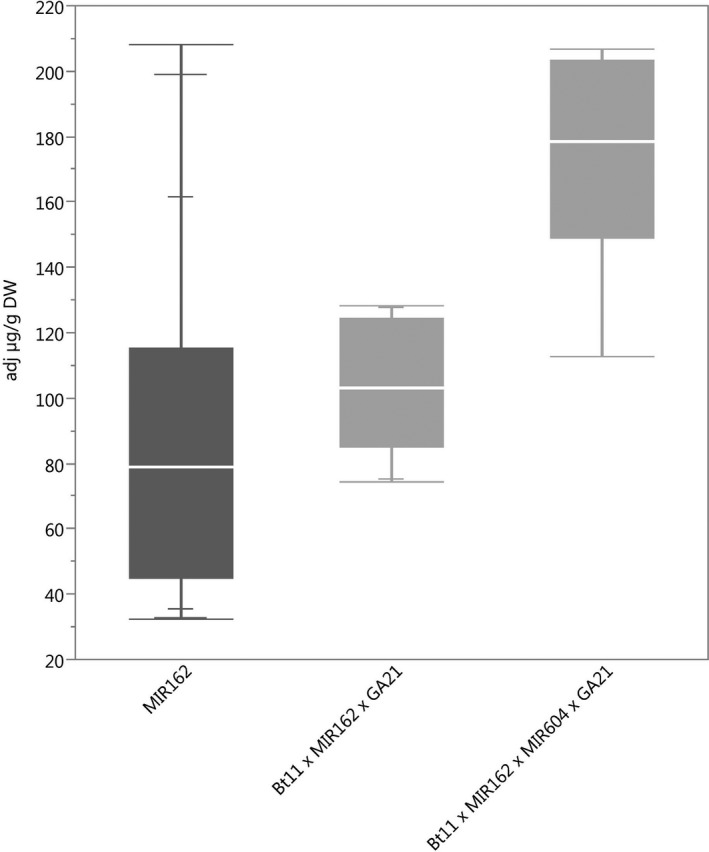
Vip3Aa20 concentrations in event MIR162 (dark grey) and stacks containing event MIR162. *N *=* *52 (MIR162), 10 (Bt11 × MIR162 × GA21) and 9 (Bt11 × MIR162 × MIR604 × GA21); Number of environments (combination of germplasm, year grown and location) = 6 (MIR162), 1 (Bt11 × MIR162 × GA21), 1 (Bt11 × MIR162 × MIR604 × GA21); × = individual datapoints for Vip3Aa20 in MIR162 maize and Bt11 × MIR162 × MIR604 × GA21 maize grown together in one field trial.

**Figure 5:**
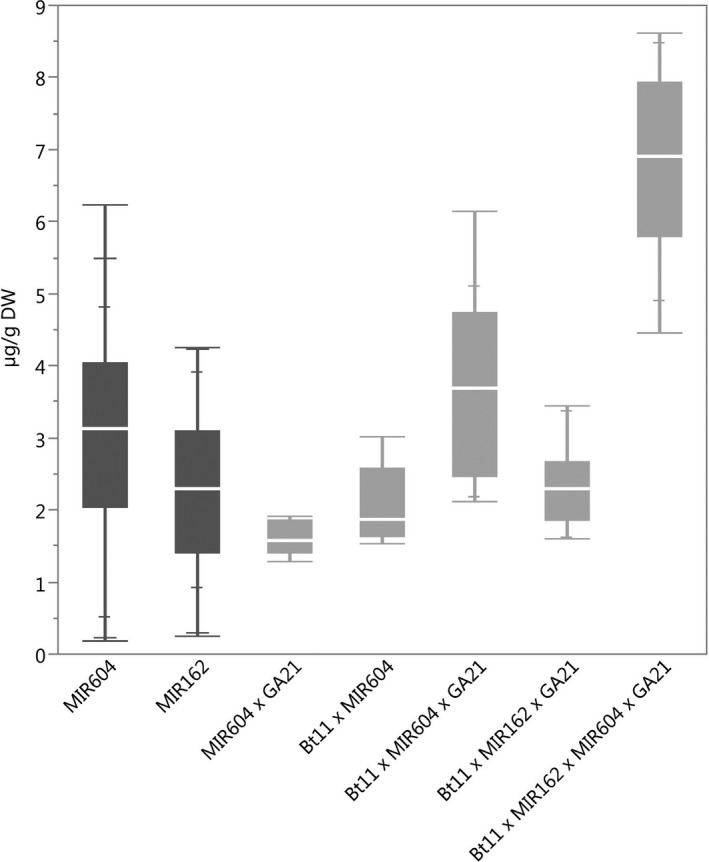
Phosphomannose isomerase concentrations in event MIR604, event MIR162 (both dark grey) and stacks containing events MIR604 and MIR162. *N *=* *101 (MIR604), 52 (MIR162), 5 (MIR604 × GA21), 5 (Bt11 × MIR604), 29 (Bt11 × MIR604 × GA21), 10 (Bt11 × MIR162 × GA21) and 17 (Bt11 × MIR162 × MIR604 × GA21); Number of environments (combination of germplasm, year grown and location) = 7 (MIR604), 6 (MIR162), 1 (MIR604 × GA21), 1 (Bt11 × MIR604), 3 (Bt11 × MIR604 × GA21), 1 (Bt11 × MIR162 × GA21) and 1 (Bt11 × MIR162 × MIR604 × GA21).

The ranges of concentration for each of the transgenic proteins in maize grain for all stacks were within the range of those for the corresponding single events (Figures 1, 2, 3, 4, 5) except for Cry1Ab in two Bt11 × MIR604 × GA21 samples, which were very slightly lower than the range in Bt11 (Figure 1). As expected, PMI concentrations in Bt11 × MIR162 × MIR604 × GA21 maize grain were higher than those in MIR162 or MIR604 due to the expression of two *pmi* genes, each contributed by MIR162 and MIR604 (Figure 5). The concentrations of Cry1Ab in Bt11 × MIR162 × GA21 and Vip3Aa20 in Bt11 × MIR162× MIR604 × GA21 were generally higher than in the other stacks (Figures 1 and 4) but were still within the ranges of expression for the single events (Bt11 and MIR162) grown in the same trials and in the same germplasm. Concentrations of PAT in event Bt11 and stacks containing Bt11 are not included because PAT concentrations were typically less than the limit of quantitation in grain. While these results do not indicate a difference in expression levels due to stacking of GM events, they do illustrate how environment and germplasm can affect protein expression levels.

### Molecular characterization

**Figure 6:**
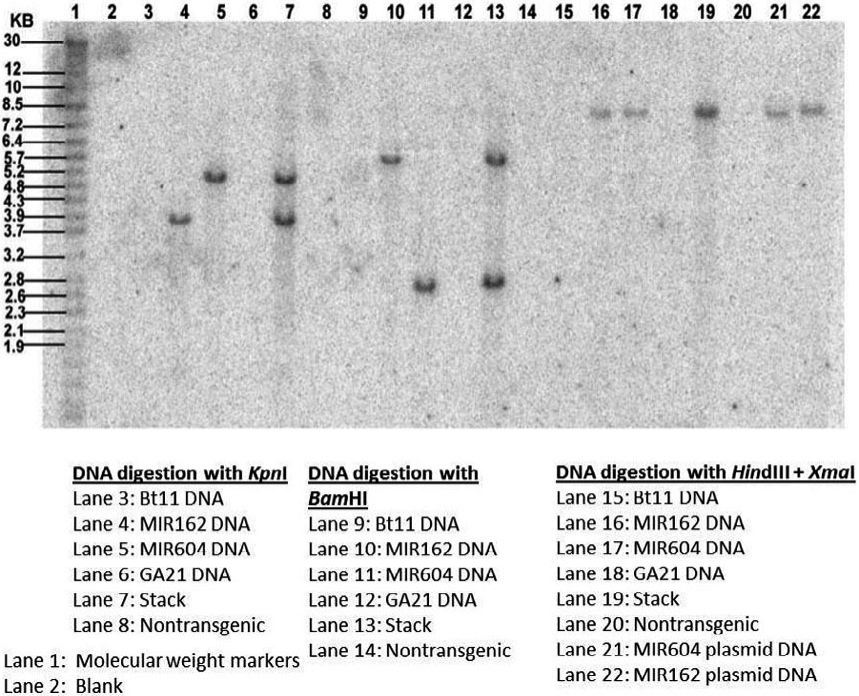
Southern blot analysis confirming presence of *pmi* in event MIR162, event MIR604 and in Bt11 × MIR162 × MIR604 × GA21.

The Southern analyses had the expected hybridization patterns of the *cry1Ab* and *pat* genes from Bt11, the *vip3Aa20* and *pmi* genes from MIR162, the *mcry3A* and *pmi* genes from MIR604 and the *mepsps* gene from GA21 contained in the stacks. An example Southern blot analysis for *pmi* is shown in Figure 6. These results demonstrated that inheritance and integrity of the transgenic inserts were preserved during conventional breeding for production of the stacks Bt11 × GA21, Bt11 × MIR604, MIR604 × GA21, Bt11 × MIR604 × GA21, Bt11 × MIR162× GA21 and Bt11 × MIR162 × MIR604 × GA21 maize.

## Discussion

The World Health Organization (WHO 1995) has stated that when two plants, which are substantially equivalent to conventional varieties are crossed by conventional breeding, the combined event product is expected to be substantially equivalent to the individual events. The composition, protein expression and molecular characterization data reported herein for several stacked GM event combinations and the component single events supported the position of the World Health Organization. Additionally, these results were consistent with the conclusions of EFSA following review of applications submitted for import approval of these single events and stacked GM event combinations (EFSA 2005, 2009a,b,c, 2010a,b,c, 2011b, 2012, EFSA, GMO Panel, 2015).

More than 20 different GM crops with stacked events have been evaluated by EFSA and other regulatory agencies and in all cases EFSA concluded that there were no compositional, agronomic or phenotypic changes that would raise safety concerns (Kok *et al*., 2014).

In considering the safety of stacked GM events for food and feed use, Steiner *et al*. (2013) posed two questions: (i) Is genomic instability increased in stacked GM events, and (ii) Can potential interactions between the transgenes and their products impact safety? An overview of genome plasticity and the mechanisms known to contribute to changes in plant genomes are well described by Weber *et al*. (2012). These molecular mechanisms naturally occur in plants, and as conventional breeding to combine GM events does not introduce additional recombinant DNA into the genome or modify the existing genomic DNA (Pilacinski *et al*., 2011), there is no biological reason for the frequency or nature of genomic instability to differ in stacked GM events. If genomic instability does occur that affects the GM sequences contributed by the individual events, the likely outcome would be a reversion to the parental phenotype. The molecular characterization data for the GM stacks described herein confirmed stable inheritance and intactness of the inserted DNA, and supports the view that combining events through conventional breeding does not increase genetic instability.

**Figure 7:**
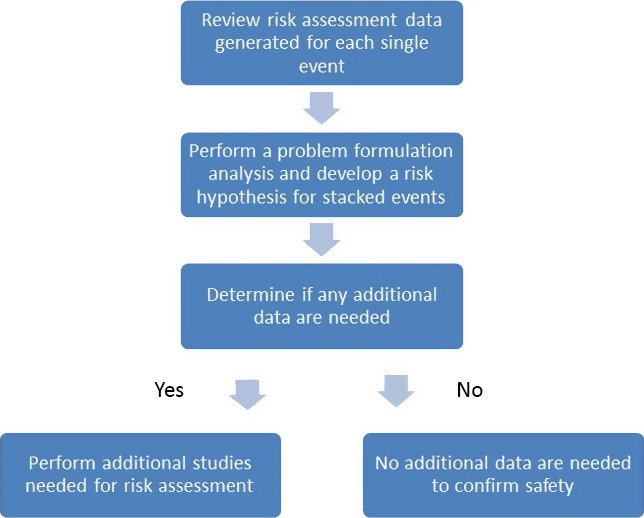
Decision process for generation of risk assessment data for stacked events.

Taken together, these data provide a strong rationale for revision of the current practices for food and feed safety assessments of stacked GM events through problem formulation focused on potential interactions resulting from the specific combination of GM events (Kok *et al*., 2014; Steiner *et al*., 2013; Weber *et al*., 2012). Problem formulation approaches to risk assessment maximize the possibility of detecting effects that indicate potential risk (Raybould, 2006). The identification and assessment of potential interactions between stacked GM events can be undertaken on a case‐by‐case basis, taking into consideration any metabolic pathways that could be influenced by the expression of the transgenes in the single events, and whether such interactions could result in changes in the plant that would impact food and feed safety. Based on each transgene's mode of action, it is possible to make predictions concerning potential interactions in the resulting GM stack. An interaction in the plant is not expected when the transgene products are not part of, or do not interact with, common metabolic pathways. Combinations of insect resistance traits fall into this category, as most insecticidal proteins have no known metabolic activity *in planta*. Herbicide tolerance is typically conferred by an herbicide‐insensitive version of the targeted enzyme or by an enzyme that metabolizes the herbicide, rendering it inactive, so it is also unlikely that a plant interaction could occur between proteins engineered for insect resistance and proteins expressed for herbicide tolerance. There are proteins that are known to act synergistically, and the potential for synergism between proteins combined by stacking of GM events should be evaluated. Although the scope of this manuscript is limited to food and feed safety, problem formulation can and should be applied to potential synergism of insect and herbicide resistance genes in assessments related to environmental safety. A diagram representing a decision‐making process for stacked GM event safety assessment data is shown in Figure 7.

Conventional breeding has a history of safe use for combining genetic information for the improvement of crop plant varieties (CLI 2011). As described herein, all new GM events undergo extensive characterization and risk assessment prior to their commercial release. Because there is no evidence of increased genome instability when GM events are stacked through conventional breeding, if there is also no plausible interaction of the transgenes or their products, then stacked GM events can be considered as safe as their component single events (CLI 2015).

## Experimental procedures

The events and stacked GM events described herein consisted of the maize events Bt11, MIR162, MIR604 and GA21, and several stacks created by combining these events through conventional breeding. Maize plants derived from event Bt11 express a truncated Cry1Ab protein for control of certain lepidopteran pests and a phosphinothricin acetyltransferase (PAT) protein that confers tolerance to glufosinate herbicide. Maize event MIR162 expresses a Vip3Aa20 protein for control of lepidopteran insect pests and a phosphomannose isomerase (PMI) protein that was used as a selectable marker. Event MIR604 expresses a modified Cry3A (mCry3A) protein for control of corn root worm and phosphomannose isomerase (PMI). Maize event GA21 expresses a modified 5‐enol pyruvylshikimate‐3‐phosphate synthase (mEPSPS) protein that confers tolerance to glyphosate herbicide. Stacked GM events were produced by combining the individual events Bt11, MIR162, MIR604 and GA21 through conventional breeding. The single events and stacks evaluated in this study were in several different genetic backgrounds.

### Compositional analysis

The single events and stacked GM events were grown in field trials to generate grain samples for compositional analysis. The field trials were conducted between 1995 and 2009 in the United States' Corn Belt, and in Europe, in agricultural regions suitable for the specific hybrids being grown. Each single event or stack was grown in separate field trials at multiple locations (Table 2). Some were grown in the same location and year, but in a different trial and in different genetic backgrounds. The field trials at each location were randomized in a complete block experimental design with three or four replicate plots at each location, except for the Bt11 trials, which consisted of a single plot at multiple locations. Developing maize ears were bagged before silk emergence to avoid pollen contamination and then self‐pollinated by hand and rebagged. The trials were maintained according to agricultural practices normally employed in the particular regions, including the use of pesticides needed to maintain plant health. Fifteen self‐pollinated ears per plot were harvested at maturity and dried to not more than 18% moisture, the grain was removed from the ears, and a well‐mixed sample from each plot of approximately 500 g was retained. The samples were stored at −20 °C ± 10 °C until they were analysed.

**Table 2:** Composition field trial years and locations

Event or stack	Years	Locations	Total no. of environments^a^
Bt11 × MIR162 × MIR604 × GA21	2006	Illinois (2), Indiana, Iowa, Minnesota, Nebraska	6
Bt11 × MIR162 × GA21	2006	Illinois (2), Indiana, Iowa, Minnesota, Nebraska	6
Bt11 × MIR604 × GA21	2006	Illinois (2), Indiana, Iowa, Minnesota, Nebraska	12
	2008	Spain (3), Romania (3)	
Bt11 × MIR604	2005	Illinois (2), Iowa, Minnesota, Nebraska, Wisconsin	6
Bt11 × GA21	2005	Illinois (3), Iowa, Nebraska, Wisconsin	12
	2008	Spain (3), Romania (3)	
MIR604 × GA21	2005	Illinois (2), Iowa, Nebraska, Wisconsin	5
MIR162	2005	Illinois (2), Iowa, Nebraska, Wisconsin	9
	2009	Illinois, Iowa, Nebraska (2)	
MIR604	2003^b^	Illinois (2), Iowa (3), Minnesota (2)	18
	2003^b^	Illinois (2), Iowa (3)	
	2008	Spain (3), Romania (3)	
GA21	2005	Illinois (3), Iowa, Minnesota, Nebraska	18
	2006	Spain (3), Romania (3)	
	2007	Spain (3), Romania (3)	
Bt11^c^	1995^b^	Iowa (2), Ohio (2), Wisconsin (2)	15
	1995^b^	Iowa (2), Ohio (2), Wisconsin (2)	
	1998^d^	France (3)	

a Environment = combination of location, year, and dgenetic background.

b The event was in two genetic backgrounds but grown at all or most of the same locations in separate field trials.

c Only one plot at each location.

d The event was in two different genetic backgrounds at one site and in a third genetic background at another site.

Levels of key nutritional components recommended by the Organization for Economic Cooperation and Development (OECD 2002) were measured in maize grain. The components measured were crude protein, fat, carbohydrates (by subtraction), acid detergent fibre, neutral detergent fibre, starch, minerals (calcium, copper, iron, magnesium, manganese, phosphorus, potassium, selenium and zinc), fatty acids (five most abundant in maize), vitamins (β‐carotene, thiamine, riboflavin, niacin, pyridoxine, folic acid and α‐tocopherol), important nutritional secondary metabolites (ferulic acid, inositol and ρ‐coumaric acid) and antinutrients (phytic acid, trypsin inhibitor and furfural).

All samples were analysed by Covance Laboratories, Inc. (Madison, WI) using published industry‐standard analytical methods (e.g. AOAC International 2005) or methods developed and validated by Covance Laboratories. The component levels were converted to equivalent units of dry weight (DW) based on the moisture content of each sample. Means, standard deviations (SD) and ranges were calculated across trials for each event or stack. Based on the compiled data for each single event, prediction intervals were calculated for the single events as a means of assessing the consistency of the data from stacks containing that single event. These prediction intervals predict with 95% confidence the range within which an additional observation would fall.

### Protein expression

Protein expression data were compiled from 26 studies including eight different field trial locations in four different countries over an eight‐year period (2002–2010) (Table 3). Comparative protein expression studies included both the stacked GM event and component single events in the same genetic background, grown in five replicate plots arranged in a randomized complete block design. In addition to the comparative protein expression studies, the single events were also grown in trials at different locations in different seasons and in different germplasm.

**Table 3:** Protein expression field trial years and locations

Event or Stack	Locations (year)
Bt11 × MIR162 × MIR604 × GA21	Illinois (2006)
Bt11	
MIR162	
MIR604	
GA21	
Bt11 × MIR162 × GA21	Illinois (2006)
Bt11	
MIR162	
GA21	
Bt11 × MIR604 × GA21	Illinois (2006), Spain (2008), Romania (2008)
Bt11	
MIR604	
GA21	
Bt11 × MIR604	Illinois (2005)
Bt11	
MIR604	
Bt11 × GA21	Illinois (2005), Spain (2008), Romania (2008), Republic of South Africa (2009)
Bt11	
GA21	
MIR604 × GA21	Illinois (2005)
MIR604	
GA21	
MIR162	Illinois (2005), Nebraska (2005), Hawaii (2008), Iowa (2009), Nebraska (2010)
MIR604	Illinois (2002), Illinois (2007), Iowa (2009)
GA21	Illinois (2004), Spain (2007 & 2008), Illinois (2007), Hawaii (2008), Florida (2006), Iowa (2009), Nebraska (2010)
Bt11	Illinois (2002), Illinois (2007), Hawaii (2008), Florida (2006), Iowa (2009), Nebraska (2010)

Each location represents a separate field trial that included all the event(s) and the stack listed in the corresponding table row.

For all protein expression studies, each grain sample consisted of all the kernels from a single ear collected at reproductive growth stage six (Abendroth *et al*., 2011). Each sample was placed immediately on dry ice upon removal from the cob and stored frozen. Grain samples were ground to a powder in the presence of dry ice, using a commercial food processor.

Proteins were extracted by homogenization in an appropriate buffer and each extract was analysed by an enzyme‐linked immunosorbent assay (ELISA) specific to the transgenic protein of interest. Concentrations of the target protein were interpolated from a standard curve then converted to μg of protein per gram of sample. Data for each transgenic protein in each single event or stack were combined across germplasm, season and location, and means, medians and ranges, displayed in box plots.

### Molecular characterization

Southern analyses were performed using standard molecular biology techniques (Chomczynski, 1992). Genomic DNA samples from each of the individual events, the stacks and nontransgenic controls were digested with restriction enzymes that resulted in a characteristic DNA hybridization pattern for each probe. For analysis of Bt11, MIR162 and MIR604 inserts, plasmid DNA representing one copy per maize genome, based on plasmid size, was included to serve as a positive control for each of the genes of interest. For the GA21 maize insert analysis, a portion of a restriction enzyme‐generated fragment containing the *mepsps* gene, representing one copy per maize genome, was used as a positive control for the *mepsps*‐specific probe. A nontransgenic control was included on each Southern blot to identify any endogenous maize sequences that could cross‐hybridize with the element‐specific probes.

## Note

1
http://www.syngenta-us.com/Agrisure/agrisure-viptera.aspx
